# Structural and Functional Analyses of a Sterol Carrier Protein in *Spodoptera litura*

**DOI:** 10.1371/journal.pone.0081542

**Published:** 2014-01-15

**Authors:** Lili Zhang, Ding Li, Rui Xu, Sichun Zheng, Hongwu He, Jian Wan, Qili Feng

**Affiliations:** 1 Guangdong Provincial Key Laboratory of Biotechnology for Plant Development, School of Life Sciences, South China Normal University, Guangzhou, China; 2 Key Laboratory of Pesticide and Chemical Biology (CCNU), Ministry of Education, College of Chemistry, Central China Normal University, Wuhan, China; University of Kentucky, United States of America

## Abstract

**Backgrounds:**

In insects, cholesterol is one of the membrane components in cells and a precursor of ecdysteroid biosynthesis. Because insects lack two key enzymes, squalene synthase and lanosterol synthase, in the cholesterol biosynthesis pathway, they cannot autonomously synthesize cholesterol *de novo* from simple compounds and therefore have to obtain sterols from their diet. Sterol carrier protein (SCP) is a cholesterol-binding protein responsible for cholesterol absorption and transport.

**Results:**

In this study, a model of the three-dimensional structure of SlSCPx-2 in *Spodoptera litura*, a destructive polyphagous agricultural pest insect in tropical and subtropical areas, was constructed. Docking of sterol and fatty acid ligands to SlSCPx-2 and ANS fluorescent replacement assay showed that SlSCPx-2 was able to bind with relatively high affinities to cholesterol, stearic acid, linoleic acid, stigmasterol, oleic acid, palmitic acid and arachidonate, implying that SlSCPx may play an important role in absorption and transport of these cholesterol and fatty acids from host plants. Site-directed mutation assay of SlSCPx-2 suggests that amino acid residues F53, W66, F89, F110, I115, T128 and Q131 are critical for the ligand-binding activity of the SlSCPx-2 protein. Virtual ligand screening resulted in identification of several lead compounds which are potential inhibitors of SlSCPx-2. Bioassay for inhibitory effect of five selected compounds showed that AH-487/41731687, AG-664/14117324, AG-205/36813059 and AG-205/07775053 inhibited the growth of *S. litura* larvae.

**Conclusions:**

Compounds AH-487/41731687, AG-664/14117324, AG-205/36813059 and AG-205/07775053 selected based on structural modeling showed binding affinity to SlSCPx-2 protein and inhibitory effect on the growth of *S. litura* larvae.

## Introduction

Sterol carrier protein 2/3-oxoacyl-CoA thiolase (SCPx), an indispensable member of SCP-2 gene family, has been found in both vertebrates and invertebrates [1]. This protein has an ability to bind diverse ligands, such as sterols, fatty acids and phospholipids, thus participating in intracellular sterol/lipid transfer processes, which affect biosynthesis and metabolism of fatty acids and sterols [2]. Insects need cholesterol for cellular membranes and ecdysteroid biosynthesis, but they lack at least two key enzymes, squalene monooxygenase and lanosterol synthase, in the *de novo* cholesterol biosynthesis pathway [3], [4]. Thus, insects must gain cholesterol or other sterols, such as the phytols, β-sitosterol, campesterol and stigmasterol from their host plants, to fulfill their sterol requirements for normal growth, development and reproduction [5]. SCP-2 protein, therefore, plays important roles in uptake and transport of sterols and fatty acids in insects [6].

In vertebrates, SCP-2 can bind both lipids and cholesterol. However, it has a higher affinity with 10–22 carbon fatty acids, especially with 14 and 16 carbon saturated fatty acids [7]. In dipteral insects, *Aedes aegypti* sterol carrier protein (AeSCP-2) can bind cholesterol [8] and palmitic acid [9], and the order (from high to low) of binding affinity for different ligands is: cholesterol, straight chain fatty acids and then kinked chain fatty acids [10]. Other AeSCP-2 like proteins, for example, AeSCP-2L2, can bind with sterols and lipids, but with higher affinities for fatty acids than for cholesterol [11]. In lepidopteran insects, such as *Spodoptera littoralis* [12] and *Manduca sexta* [13], SCPx protein has been also found to be able to bind with cholesterol and lipids.

Although members of the SCP-2 family has been identified in a broad range of organisms from bacteria to plants and mammals [2], the diverse biological functions of the SCP-2 protein family remain to be clarified. In addition, detailed relationships of the structure and function of the SCP-2 proteins are unclear, partially due to lack of definitive 3-dimensional protein structures. So far, the crystal structures of several members of the SCP-2 family have been obtained and analyzed, including *Thermus thermophilus* SCP-2 (bacterium TtSCP-2) [14], *Phytophthora cryptogea* SCP-2 (fungus PcSCP-2) [15], *Aedes aegypti* SCP-2 or SCP-2-like proteins (mosquito AeSCP-2, AeSCP-2L2 and AeSCP-2L3) [6], [9], [16], *Homo sapiens* SCP-2 (human HsSCP-2 and HsMFE-2) [17]–[18], and *Oryctolagus cuniculus* SCP-2 (rabbit OcSCP-2) [19]. Among them, the crystal structure of mosquito *Ae*SCP-2, *Ae*SCP-2L2 and *Ae*SCP-2L3 were obtained with palmitate substrate binding to the proteins [2]. The structure of human *Hs*MFE-2 SCP-2 domain was obtained with a detergent molecule TritonX-100 in its hydrophobic pocket [18]. The 3-D structure of human *Hs*MFE-2 SCP-2 domain appears similar to that of the dipteral mosquito *Ae*SCP-2, with a major difference in a loop present in the mosquito *Ae*SCP-2, which coordinates the carboxylate group of the fatty acid substrates. In mammalian proteins, this loop is replaced by a short α-helix. In addition, *Ae*SCP-2 protein exhibits a layer of four helices in the front that cover the five strands of β-sheets which binds the ligand palmitic acid by forming hydrogen bonds with arginines, and the hydrophobic binding pocket of the protein does not extend to the surface of the protein [9]. However, the human *Hs*MFE-2 SCP-2 domain binds with the substrate TritonX-100 in a horizontal direction, with the polyoxyethylene tail piercing to the exit of the binding pocket, where surrounds of a cluster of exposed hydrophobic amino acid residues [9]. Although the palmitate-ligated structures of *Ae*SCP-2 [9] or SCP-2 like proteins [6], [9], [16] have been reported, the crystal structure of the protein alone has not been obtained.

By using NBD-cholesterol fluorescence replacement assay [20], five potential chemical inhibitors (SCPIs) of *A. aegypti* SCP-2 proteins were obtained and these AeSCPIs can cause high levels of mortality in *A. aegypti*, especially for AeSCPI-1, which kill the larvae within 3 days post the treatment, whereas AeSCPI-2 caused death at larva-to-pupa transition period [20]. They also effectively reduce cholesterol uptake and cholesterol accumulation in the midgut and in fat body of *M. sexta* larvae and SCPIs are lethal to *M. sexta* larvae [13]. SCPI-1 is also lethal to *H. armigera* neonates [21]. Homology modeling 3-D structures of *Euphorbia lagascae* SCP-2 and *Arabidopsis thaliana* SCP-2 are also used to reveal the binding of the protein to diverse lipids [22]. Site-directed mutagenesis for ligand selectivity analysis reveals that a single Leu-Met exchange enhances sterol transfer activity [23]. Changing Leu99 to Met99 was sufficient to convert *E. lagascae* SCP-2 into a sterol-sensitive protein, and correspondingly, changing Met100 to Leu100 abolished the sterol sensitivity of *A. thaliana* SCP-2 [23]. In AeSCP-2, changing Phe32 to Trp32 caused significant changes in the NBD-cholesterol binding affinity and both W44E and M90L abolished the ability of binding with cholesterol but retained palmitic acid-binding capacity [24].

In the previous study, we reported identification of a SCP gene (*SlSCPx*) from *S. litura* and found that this gene has higher expression levels during the feeding stage of larvae than other stages. Knocking down this gene by RNAi suppresses the absorption of cholesterol and the development and metamorphosis of the insect [25]. We also found that *SlSCPx* is responsible for the uptake of cholesterol into the prothoracic glands where the cholesterol is used for ecdysteroid synthesis during molting and metamorphosis (unpublished data). In this study, to further investigate the binding affinity and specificity of the SlSCPx-2 protein with different sterols and fatty acids and the relationship between the structure and function, ANS fluorescent replacement assay [26] was used to screen and test optimal ligands for the protein. Additionally, a 3-D structure of SlSCPx-2 was built by Swissmodel with the human SCP-2 domain as a template. Several novel potential compounds which can bind to SlSCPx-2 were identified from the LipidBank and SPECs databases by using structure-based virtual screening strategy [27]. Furthermore, the hit compounds were chosen as probe molecules and their probable interactions with the individual residues of the protein have been examined by jointly using the molecular docking and site-directed mutagenesis approaches.

## Materials and Methods

### Rearing of insects

*Spodoptera litura* (Lepidoptera:Noctuidae) insect was provided by the Entomology Institute of SUN YAT-SEN University, Guangzhou, China. Larvae were reared in artificial diet (soybean powder: 100 g, wheat bran: 80 g, yeast: 26 g, casein: 8 g, Vitamin C: 8 g, choline chloride: 1 g, sorbate: 2 g, cholesterol: 0.2 g, inositol: 0.2 g, agar: 26 g and formaldehyde: 2 ml in 1 liter) at 26°C, 70–80% humidity and a photoperiod of 12 h light and 12 h dark until they reached adult moths.

### Chemicals and molecule simulation softwares

1, 8-Anilino-1-naphthalenesulfonic acid ammonium salt (1, 8-ANS), cholesterol, stearic acid, palmitic acid, oleic acid, linoleic acid, arachidonate acid, stigmasterol and ergosterol were purchased from Sigma-Ardrich (Shanghai, China). Stock solutions of these lipids and sterols were dissolved in ethanol and stored at −20°C. The AeSCPI-1 and AeSCPI-2 were provided by Dr. Que Lan in Department of Entomology, University of Wisconsin-Madison, Madison, USA. *Escherichia coli* strain DH5α was maintained in the laboratory.

The three dimensional crystal structure of human *Hs*MFESCP-2 was obtained from the Protein Data Bank (PDB) (www.rcsb.org/pdb/; PDB ID: 1IKT). Amino acids in the active site of *Hs*MFESCP-2 were from Val11 to Ile36, which was confirmed with the Pocket Finder (http://www.modelling.leeds.ac.uk/pocketfinder/). Fatty acids and sterols are downloaded from the database of Japanese Conference on the Biochemistry of Lipids named LipidBank (JCBL, http://lipidbank.jp/). SURFLEX module of SYBYL-7.3 program package was used for virtual docking analysis. Molecular dynamic (MD) study was performed by using SANDER module of AMBER8.0 package. All calculations were performed on a CCNUGrid-based computational environment (CCNU-Grid Web site http://202.114.32.71:8090/ccnu/chem/platform.xml).

### Homology modeling, molecular dynamics simulation and docking-based virtual screening

In view of the sequence alignment, a 3-D structure of SlSCPx-2 was built by using the SWISSMODEL server (Automated Comparative Protein Modeling Server, Version 3.5, Glaxo Wellcome Experiment Research, Geneva, Switzerland) and the X-ray crystallographic structure of *H. sapiens Hs*MFESCP-2 (Protein Data Bank ID: 1IKT) as a template. The *Hs*MFESCP-2 has 39% sequence identity with the target protein. All hydrogen atoms were subsequently added to the unoccupied valence of heavy atoms of the modeled SlSCPx-2 at the neutral state by using the BIOPOLYMER module of the SYBYL 7.3 program package.

To obtain optimal modeling and 3-D conformation of the complex of SlSCPx-2-TritonX, a molecular dynamic (MD) study was further performed by using SANDER module of AMBER8.0 package. The leaprc.ff99 force field parameters were loaded for the holo-protein system and a set of default parameters provided by the AMBER8.0 was adopted for the non-natural TritonX-100. The system was neutralized first by adding Na^+^ ions and then solvated into an octahedral box of TIP3P water molecules. Besides, before starting the production-run phase, the following equilibration protocol was employed. First, all water molecules of the TIP3P box were minimized 2000 steps by steepest descent and 2000 steps by conjugate gradient, respectively, while the holo-protein system was frozen. Then, the whole system (holo-protein plus water box) was minimized 4000 steps by using amber force field with releasing the whole system. Finally, the whole system was slowly heated from 0 to 300 K over 100 ps before MD simulation. Trajectories were recorded every 1 ps during the entire MD simulation process [28]. A modeling averaged conformation was derived from the trajectories of the converged 32810–33600 ps. Then the docking strategy described in the next part was employed to get a reasonable SlSCPx-2-SCPI1 complex. Another time MD simulation process was used and a modeling averaged conformation was derived from the trajectories of the converged 18900–20900 ps and subjected to a subsequent minimization using Tripos force field of SYBYL7.3 with a rms gradient of 0.05 kcal/(mol·Å) to generate the final theoretically reasonable 3D modeling conformation of SlSCPx-2 for subsequent virtual screening. The constructed structure was evaluated by the program SIRIUS (version 1.2).

In order to detect the interaction mechanism and illustrate the accurate binding model for the active site of SlSCPx-2 with its potential ligands, molecular docking analysis was carried out by using SURFLEX module of SYBYL-7.3 program package on the basis of the target structure optimized by MD methods by using SANDER module of Amber8.0 package. The .protomol parameters were tuned with a proto_threshold of 0.3 and proto_bloat of 2 and dockings were carried out using 20 initial conformations for each ligand using these protomol parameters choice. The Surflex-Dock scoring function was used in these docking process.

By using the similar docking process, the structure-based virtual screening of compounds from the LipidBank and SPECs database (http://lipidbank.jp/; http://www.specs.net/snpage.php?snpageid=home) was performed. The virtual screening strategy adopted in the present study consisted of one step of 2-D ligand-based searching in terms of Lipinski rules and two steps of 3-D receptor–ligand binding mode-based molecular docking evaluations for the hit compounds listed in SPECs database. On the first step of 2-D ligand-based searching, the criteria of the Lipinski rules (≤5 H-bond donors, no. of OH and NH groups; ≤10 H-bond acceptors, no. of O or N atoms; MW≤500 Da, M log P≤5) was employed to preselect all the molecules in the SPECs database. Other default parameters were adopted in SURFLEX docking and virtual screening. All calculations were performed on a CCNUGrid-based computational environment (CCNUGrid Web site: http://202.114.32.71: 8090/ccnu/chem/platform.xml).

### Expression and purification of SlSCP-x and SlSCPx-2 recombinant proteins

SlSCP-x and SlSCPx-2 recombinant proteins were expressed in the DH5α/pPROEXTM HTa host/vector expression system (Life Technologies, Burlington, Canada). The open reading frames (ORFs) of the *SlSCPx* and *SlSCPx-2* cDNAs were amplified by PCR and inserted into the pPROEXTM HTa expression vector between the *Eco*R I and *Not* I sites with 6× His tag on the *C*-terminal ends of the target sequences. *E. coli* cells (DH5α) were transformed with the recombinant plasmid DNAs (pPROEXTM HTa-*SlSCPx* and pPROEXTM HTa-*SlSCPx-2*). Expression of the His-tagged SlSCPx and SlSCPx-2 fusion proteins were induced by adding IPTG (isopropyl-β-D-Thiogalactopyranoside) at a final concentration of 0.4 mM. The recombinant proteins were purified using His-tag affinity columns according the manufacturer's instruction (Novagen, Darmstadt, Germany). Purified recombinant proteins (rSlSCP-x and rSlSCPx-2) were dialyzed using Amicon-Ultra-4 Ultrafiltration tube (Millipore, Germany) against 1, 8-ANS reaction buffer (25 mM Na_3_PO_4_, 75 mM NaCl, 0.1 mM EDTA; pH 7.0). The proteins were then concentrated to 100 µM in reaction buffer for further use.

### Fluorescence binding and displacement assay

Binding of the fluorescent probe 1, 8-ANS to sterol carrier protein (SlSCPx-2) is measured by relative increase in fluorescent intensity [26]. Steady-state fluorescence spectra were measured on a Cary Eclipse fluorescence spectrophotometer (Varian, Mulgrave, Australia) using a 1-cm path length cuvette. The binding was monitored by measuring the fluorescence signal between 400 and 600 nm following excitation at 350 nm. Slit widths were set to 5 and 10 nm for the excitation and emission monochromators, respectively. To assess the binding affinity for SlSCPx-2, 1, 8-ANS at various concentrations from 0 to 90 µM was titrated into a diluted solution of 50 µM SlSCPx-2. All measurements were performed at room temperature and the samples were equilibrated for 5 min prior to measurement. At each of the given concentrations, at least three replicates were performed.

Binding of the fluorescent probe 1, 8-ANS to SlSCPx-2 can be competed with physiological ligands [22]. SlSCPx-2 at 15 µM was incubated with 1, 8-ANS at 15 µM in the reaction buffer (pH 7.0) at room temperature for 2.5 min. After the incubation, each potential ligand at a serial of concentration (0–100 µM) was added into the 1, 8-ANS-protein mixture for another 2.5 min and the fluorescence intensity of the 1,8-ANS-protein complex was measured as previously described. The control was the reaction of ANS probe and a series of concentration (0–100 µM) of ligands without the protein. To calculate binding activity of the ligands and the protein, the fluorescence intensity of the controls were subtracted from the total fluorescence intensity of the 1, 8-ANS-protein complex in the presence of ligands tested. Each ligand replacement assay was repeated for three times. For ligand competition assays with 1, 8-ANS in the presence of rSlSCPx-2, the 50% effective concentration (IC_50_) was obtained using a single-site competition, nonlinear regression model in GraphPad Prism version 4.0 (GraphPad Software Inc.) using the equation:
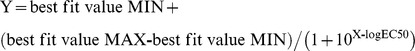



### Site-directed mutagenesis of SlSCPx-2 and bio-affinity assay

Various mutations in His-tagged SlSCPx-2 were constructed by using the Stratagene QuickChange Site-Directed Mutagenesis Kit (TAKARA, Dalian, China). Recombined plasmid containing SlSCPx-2 fused with the His-Tag sequence in a pPROEXTM HTa vector was used as a template. Two complementary primer pairs containing the desired mutations were used to construct each gene mutant. All mutant sequences were confirmed by DNA sequencing. The binding affinity of the mutants with the various ligands was examined using the fluorescence displacement assay as described above.

### Bioassays

Newly hatched *S. litura* larvae (30 larvae per treatment) were reared in artificial diet in presence of each chemical at different concentrations of 3, 12 and 48 µg/g diet. Control larvae were fed in the diet with diluted acetone (solvent for the chemical). Fresh diet was provided every other day. Larvae development was monitored until pupation. Larvae were weighted at 13 days post egg hatching when most of the larvae were at 5^th^ instar stage.

## Results

### Homology modeling, molecular dynamics simulation

**Figure 1:**
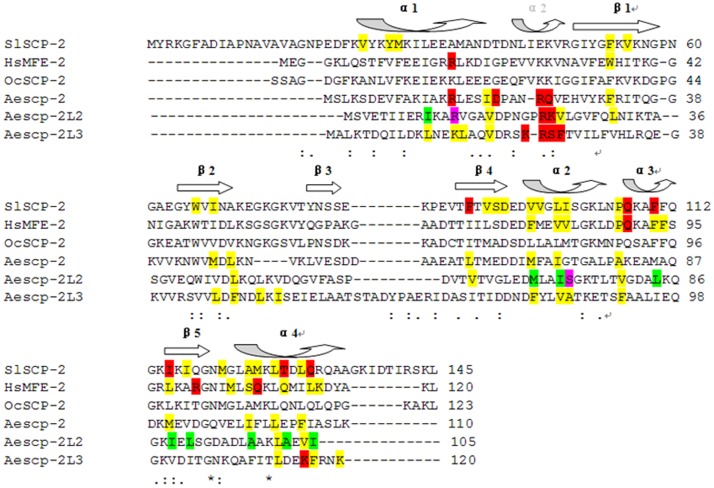
Amino acid sequence alignment of SlSCPx-2 with the SCP-2s, the 3-dimensional structures of which are known. Red colored residues: coordinating with the polar groups of the ligand. Purple colored residues: coordinating with the polar groups of the second ligand in one chain. Yellow colored residues: interacting directly with the hydrophobic groups of the ligand. Green colored residues: interacting directly with the hydrophobic groups of the second ligand in another chain. Secondary structures are indicated above the amino acid sequences, whereas the secondary structures different from mosquito SCP-2s are in dotted arrow. The alignment was prepared with the program CLUSTALW2 online (http://www.ebi.ac.uk/Tools/msa/clustalw2/).

A 3-D structure of SlSCPx-2 was established by SWISSMODEL against human SCP-2 like protein, HsMFESCP-2 (PDB code 1IKT), whose crystal structure was obtained with a TritonX-100 molecule in its hydrophobic ligand binding cavity [17]. The reason for use of this protein as a template is that structural alignment of the sequences shows that the 3-D structure of SlSCPx-2 shares 39% identity to MFESCP-2, the highest between SlSCPx-2 and the known SCP-2 proteins, except the *Oryctolagus cuniculus* SCP-2 (rabbit OcSCP-2), which has no ligand in the binding pocket [19], including those proteins, such as *Thermus thermophilus* SCP-2 (bacterium TtSCP-2) [14], *Phytophthora cryptogea* SCP-2 (fungus PcSCP-2) [15], *Aedes aegypti* SCP-2 or SCP-2-like proteins (mosquito AeSCP-2, AeSCP-2L2 and AeSCP-2L3) [6], [9], [16] and *Homo sapiens* SCP-2 (human HsSCP-2 and HsMFESCP-2) [17]. Identities of SlSCPx-2 amino acid sequence to HsMFESCP-2, OcSCP-2, AeSCP-2, AeSCP-2L2 and AeSCP-2L3 are 39, 44, 28, 17 and 10%, respectively (Fig. 1). Lepidopteran SlSCPx-2 has a higher identity to the mammal SCP-2 than to Dipteral mosquito SCP-2 proteins. Usually, when the protein structures share a 25% identity, the accurate modeling of 3-D structure can be achieved [29]. In this modeling the temple HsMFESCP-2 and SlSCPx-2 share 39% identity, suggesting that our homology modeling result would be reliable.

To obtain a stable and optimal structure for the next virtual screening, docking analysis of inhibitors of AeSCP (including *Ae*SCPI-1, -2, -3, -4 and -5), which have been demonstrated to bind with AeSCPs by using NBD-cholesterol competitive binding assays [26], was carried out by using SURFLEX module of SYBYL-7.3 based on the target structure built by SWISSMODEL. In this analysis, modeling structures of SlSCPx-2/AeSCPIs complex were used because no crystallographic structure of SlSCPx-2/cholesterol complex is available. The docking result indicated that AeSCPI-1 had the highest docking score (Table 1). Therefore in the following molecular dynamic (MD) analysis the SlSCPx-2/AeSCPI-1 complex structure was used.

**Table 1:** Docking analysis of SlSCPx-2 with AeSCPIs and cholesterol.

Compounds	Docking score	Compounds	Docking score
AeSCPI-1	9.27	AeSCPI-2	6.41
AeSCPI-5	9.15	AeSCPI-3	5.29
Cholesterol	8.41	AeSCPI-4	2.87

**Figure 2:**
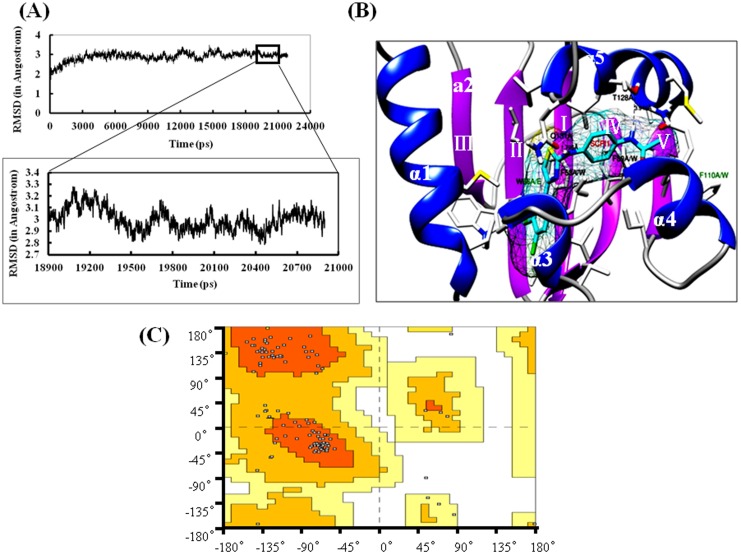
Modeling of SlSCPx-2 protein. (A) Dynamics curve. Trajectories were recorded every 1ps during the entire MD simulation process. Each point represents a 3D-structure of SlSCPx-2/SCPI1. The final optimal structure SlSCPx-2/SCPI1 was an averaged conformation modeling derived from the trajectories of the converged 18900–20900 ps. (B) The final optimal structure of the SlSCPx-2/SCPI1 complex in a ribbon view. The α–helixes are shown in blue and the β–sheets are shown in pink. Selected amino acid residues that directly interact with the bound ligand (black colored sticks; F53, F89, T128, and Q131) and indirectly contact with the ligand (dark green colored stick; W66 and F110) were used for point mutation. The AeSCPI-1 is highlighted as stick model colored cyan. (C) The Ramachandran plot of the SlSCPx-2/SCPI1 complex. The orange color represents those residues in the most favored regions; The dark yellow represents those residues in the additionally allowed regions; The light yellow represents those residues in generously allowed regions; The white represents those residues in disallowed regions.

To obtain reasonable 3-D configuration of SlSCPx-2, MD analysis was further performed using the SlSCPx-2/AeSCPI-1 complex by using SANDER module of AMBER8.0 package. The MD optimization result of the SlSCPx-2 protein showed that the cavity is too small to accommodate any lipids or sterols (data not shown). It has been reported that ligand binding flexibility of human SCP-2-like protein can be increased by enlarging the hydrophobic binding pocket [17]. In another word, ligand binding in the hydrophobic binding pocket is flexible to accommodate different potential ligand. To optimize the structure and allow potential ligands to fix in the pocket, the protein-ligand complex (SlSCPx-2/AeSCPI-1), rather than the protein alone, was then used so that the binding pocket can be enlarged and stabilized by AeSCPI-1 during the MD optimization for ligand binding in the subsequent virtual ligand screening process. The rmsd for all atoms of the SlSCPx-2/AeSCPI-1 complex over the simulation time was determined by using PTRAJ module. The plot of the evolution of rmsd with optimization time is shown in Figure 2A. The whole system achieved a dynamics convergence at around 18900–20900 ps, from which an averaged conformation was derived. An optimized structure of the SlSCPx-2/AeSCPI-1 complex after MD analysis is shown in Figure 2B. Ramachandran plot analysis further confirmed the quality of the MD-based optimized 3-D structure of the SlSCPx-2/AeSCPI-1 complex (Fig. 2C). In this structure 92.85% of the residues were distributed in the most favored regions, 7.14% in the additional allowed regions, and no residues in the disallowed regions. Thus, a reliable 3-D structure of the SlSCPx-2/AeSCPI-1 complex was obtained. In this modeled structure (Fig. 2B), the protein consists of a five stranded β-sheets and five α-helices in an order of α1-α2-βI-βII-βIII-βIV-α3-α4-βV-a5. The five α-helices are around the β-sheets. A cavity as the ligand binding site exists at the interface between the α-helices and the β-sheets, in which AeSCPI-1 sits. Except α2-helix and βIII-sheet, all of other helices and sheets contribute to the ligand binding in the pocket and those amino acid residues that have direct or indirect interaction with the AeSCPI-1 ligand lie on these α-helices and the β-sheets, such as F53, W66, F89, F110, I115, T128 and Q131.

**Figure 3:**
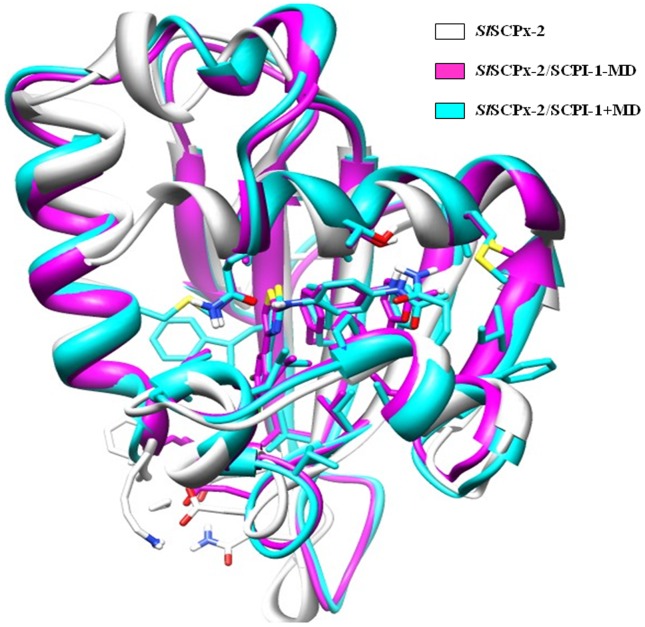
Superposition of modeling single structure of SlSCPx-2 before optimization by using MD simulation (SlSCPx-2, white ribbon) with modeling structure of SlSCPx-2/AeSCPI-1 complex optimized before using MD simulation (SlSCPx-2/AeSCPI-1−MD, magenta) and modeling structure of SlSCPx-2/AeSCPI-1 complex optimized by using MD simulation (SlSCPx-2/AeSCPI-1+MD, cyan ribbon).

To compare the difference or similarity of the three 3-D structures (SlSCPx-2 alone, SlSCPx-2/AeSCPI-1 complex before MD optimization, referred SlSCPx-2/AeSCPI-1−MD, and SlSCPx-2/AeSCPI-1 complex after MD optimization, referred SlSCPx-2/AeSCPI-1+MD), superposition analysis was performed. The results indicated that the three configurations were very similar to each other, with the biggest difference of 1.165 Å between SlSCPx-2 and SlSCPx-2/AeSCPI-1−MD, while the difference between SlSCPx-2 and SlSCPx-2/AeSCPI-1+MD was 1.122 Å, and the difference between SlSCPx-2/AeSCPI-1−MD and SlSCPx-2/AeSCPI-1+MD was 0.623 Å (Fig. 3). This implies that the compound AeSCPI-1 can stabilize and enlarge the ligand-binding cavity after MD optimization, but not change the spatial configuration of the protein. Thus, a high quality and stable 3-D structure of the SlSCPx-2/AeSCPI-1 complex was made available for use in the docking-based virtual screening for SlSCPx-2-binding compounds.

### Screening of SlSCPx-2 binding compounds

To screen potential compounds that can bind to SlSCPx-2, a set of screening processes were performed using the above SlSCPx-2/AeSCPI-1 complex model (Fig. 2B). Firstly, 462 sterols and 731 fatty acids from LipidBank were docked with the protein structure by Surflex module of SYBYL-7.3 package. In the docking process, most of the sterols that had complicated side groups, such as digitogenin, scilliroside, escin, echujin, had been screened out because they have complex side chains and could not be fixed in the binding cavity. Only those compounds which have a standard cut-off docking score of 5 were considered as potential ligands. Therefore, ca. 10% (54/462) of the sterols that have highest scores were chosen to determine their affinities to SlSCPx-2 and many of them are naturally present in plants, such as α-spinasterol, lumisterol, campesterol, stigmasterol, β-sitosterol, chondrillasterol, spinasterol and crinosterol (Table 2) [30]. The protein had high binding affinities to fatty acids, particularly the mediate and long chain fatty acids, such as 14–16C fatty acids and 20–22C fatty acids (Table 3), accounted for 10% of the total docking potential ligands [30]–[31]. Some of these fatty acids are commonly found in plants, such as trans-brassidic acid, palmitic acid, stearic acid, oleic acid, linoleic acid and arachidonic acid.

**Table 2:** Some samples of sterols with relatively high affinities (docking score) to SlSCPx-2 protein.

Sterols	Docking score	Sterols	Docking score	Sterols	Docking score
Epicholestenol	11.64	Desmosterol	6.81	Fungisterol	6.14
Nandrolone	8.91	Avenasterol	6.81	24-Epifungisterol	6.12
β-ecdysone	8.52	α_1_-Sitosterol	6.77	Fucosterol	6.14
α-Spinasterol*	8.48	Isocitrostadienol	6.73	a_1_-Sitosterol	6.12
Clerosterol	8.47	Peposterol	6.65	β-Ergostenol	6.12
Cholesterol *	8.41	Coprostane	6.58	Citrostadienol	6.10
7-Dehydrositosterol	8.03	γ-ergostenol	6.51	7-keto-campesterol	6.09
Crinosterol	8.00	Chalinasterol	6.42	22,23-Dihydrobrassicasterol	6.08
β-Sitosterol*	7.82	25-hydrochondrillasterol	6.41	Campesterol*	6.05
Bufotoxin	7.79	Episterol	6.40	Methenolone	6.04
5β,6β-epoxycholesterol	7.69	Squalamine	6.39	Ergosterol *	5.98
γ-Sitosterol	7.61	α-ergostenol	6.37	7α-hydroxycholesterol	5.94
5-Stigmasten-3-one	7.56	24,25-dimethyl-5α-cholesta-7,24(28)-dien-3β-ol	6.31	7β-hydroxycholesterol	5.94
7-Keto-β-sitosterol	6.97	22-Dihydrochondrillasterol	6.27	Lumisterol*	5.89
5,6-epoxy-β-sitosterol	6.97	9,11-Dehydroergosterol	6.21	9β-Ergosterol	5.89
Spinasterol*	6.91	Neoergosterol	6.18	Coprostenol	5.87
Chondrillasterol*	6.90	4-stigmastene-3,6-dione	6.16	5β-cholestane-3α,7α,12α-triol	5.83
Dehydroergosterol	6.89	Stigmasterol *	6.16	Cycloeucalenol	5.82

These sterols are commonly found in plants.

**Table 3:** Some samples of fatty acids with relatively high binding affinities (docking score) to SlSCPx-2 protein.

Fatty acids	Docking score	Fatty acids	Docking score
Nonacosanoic acid	11.32	20-hydroxyeicosanoic acid	10.49
Mycoceranic acid	11.30	Cis-erucic acid	10.46
11,12-dihydroxyarachidic acid	11.58	Methyl-10,12-Dihydroperoxy-8,13,15-Octadecatrienoate	10.45
Methyl 9-butylperoxy-10,12-octadecadienoate	11.46	Arachionic acid	10.31
Trans-brassidic acid	11.43	11S-hydroxy-5Z,8Z,12E,14Z-eicosatetraenoic acid	10.22
4, 8, 12, 15, 19, 21-tetracosahexaenoic acid	11.37	Timnodonic acid	10.11
12-oxo-5Z,8Z,10E,14Z-eicosatetraenoic acid	11.30	4-hydroxystearic acid	10.10
(+)-24-methylhexacosanoic acid	11.21	9,10-dihydroxystearic acid	10.10
21-hydroxyheneicosanoic acid	11.20	Tricosanoic acid	9.59
Sativic acid	11.20	Methyl 11-Hydroperoxy-5,8,12,14,17-icosapentaenoate	9.46
Isopentacosanoic acid	11.12	3, 13, 19-Trimethyltricosanoic acid	9.39
2-hydroxybehenic	11.10	2-hexadecenoic acid	9.22
Isobehenic acid	11.09	Hydroxynervonic acid	9.13
Heneicosanoic acid	11.07	4,16-dimethyloctadecanoic acid	9.03
Trans-selacholeic acid	11.05	Trans-5, cis12-octadecadienoic acid	8.66
15-hydroxystearic acid	11.03	19-hydroxynonadecanoic acid	8.54
Lumepueic acid	11.03	Stearic acid	8.53
cis-Dotriacontenoic acid	11.01	Oleic acid	8.50
2-Hydroxybehenic	10.92	9-Hexacosenoic acid	8.46
9-hydroxy-10,12-octadecadienoic acid	10.88	Palmitic acid	8.34
10-ethoxy-9,13-dihydroxy-11-octadecenoic acid	10.69	Linoleic acid	8.31
Trans-brassidic acid	10.56	Nervonic acid	7.92
Sterculic acid	10.55	Lignoceric acid	7.21

### Fluorescence binding and displacement assay

**Figure 4:**
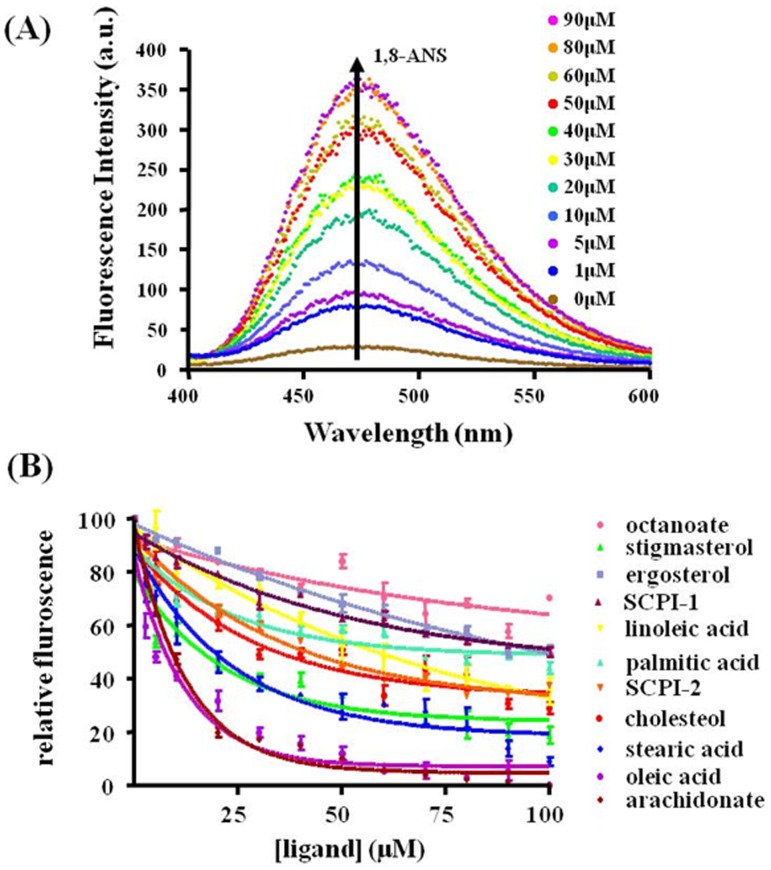
Displacement assay of 1, 8-ANS bound to SlSCPx-2 by different ligands. (A) Normalized fluorescence spectra of SlSCPx-2 bound 1,8-ANS with the increase of the 1, 8-ANS concentrations from 0 to 90 µM when the SlSCPx-2 concentration was fixed at 50 µM, showing the increase of the fluorescence intensities. (B) Displacement assay of 1, 8-ANS bound to SlSCPx-2 by selected compounds. 1, 8-ANS at 15 µM was pre-incubated with 15 µM SlSCPx-2 and then was displaced with different ligands, respectively. The loss of the 1, 8-ANS fluorescence signal was observed with the addition of increasing concentrations of octanoate (pink diamonds), cholesterol (red diamonds), arachidonic acid (brown diamonds), stearic acid(blue diamonds), linoleic acid (yellow triangles), stigmasterol (green triangles), ergosterol (light blue), oleic acid (purple), palmitic acid (light green), AeSCPI-1(dark red) and SCPI-2 (orange), respectively. Excitation at 350 nm; Emission, 475 nm.

1, 8-ANS has been used as a sensitive probe for examining hydrophobic ligand binding by providing a substantial fluorescence enhancement upon binding to some proteins [32]. In the present assays, when the concentration of the SlSCPx-2 protein was fixed at 50 µM and the concentrations of 1, 8-ANS were increased from 0 to 90 µM, the fluorescence intensities were increased correspondingly (Fig. 4A), indicating that 1, 8-ANS bound to the protein. This result indicated that within the range between 0 and 90 µM, the fluorescence intensity increases with binding activity of the protein to 1, 8-ANS and this assay can be used for replacement assay for various ligands.

To determine whether or not the results derived from the *in silico* docking assay (Tables 2 and 3) is coincident with those from the fluorescence displacement assay, some of the compounds that exist in plants and can bind to SlSCPx-2 with high docking scores, such as stigmasterol, ergosterol, cholesterol, palmitic acid, stearic acid, oleic acid, linoleic acid and arachidonic acid, were selected to be confirmed by the fluorescence displacement assay. Octanoate that had much lower docking score (data not shown) was chosen as negative control. Two inhibitors of *A. aegypti* SCP-2 (AeSCPI-1 and AeSCPI-2) [20] were also tested for their binding affinity with SlSCPx-2. These two compounds have been found to be able to inhibit cholesterol absorption in *S. litura* (unpublished data) and they had high docking scores with the protein in the docking assay (Table 1). Displacement of 1, 8-ANS by different sterols and fatty acids was measured when increasing amounts of the tested ligands were added to the reaction system. The result revealed that SlSCPx-2 could bind with fatty acids in a similar order (Fig. 4B; Table 4) as in the docking assay (Table 3), except for stearic acid and oleic acid. For sterols, slight differences were found between the results of the *in silico* docking score (Table 2) and the *in vitro* assay (Fig. 4B; Table 4). For example, cholesterol had a lower docking score than SCPI-1 (Table 2), but it had a higher affinity than SCPI-1 in the *in vitro* docking assay (Table 4). Stigmasterol had a higher docking score than ergosterol, but stigmasterol has a lower binding affinity than ergosterol. However, theoretical and experimental results are generally agreed with each other. This agreement indicating that the modeling strategies and screening processes used in this study are valid and promising for searching novel lead compounds binding to SlSCPx-2. These results also suggest that SlSCPx can bind not only sterols but also fatty acids.

**Table 4:** Inhibition effects (IC_50_) of different ligands in replacement assay of the 1, 8-ANS-bound SlSCPx-2.

Ligands	IC_50_ (µM)	RBA(%)	Ligands	IC_50_ (µM)	RBA (%)
Arachidonate	18.12±0.83	277	Linoleic acid	143.00±0.99	35
Oleic acid	24.37±1.14	206	AeSCPI-1	160.90±5.03	31
Stearic acid	39.82±1.17	126	Ergosterol	167.60±0.34	30
Cholesterol	50.13±0.80	100	Stigmasterol	189.60±2.29	26
AeSCPI-2	55.03±0.66	91	Octanoate	217.90±1.53	23
Palmitic acid	59.05±1.29	85			

IC_50_: the concentration at 50% inhibition and the data are derived from assays described in Fig. 4B. RBA: relative binding affinity. It was calculated by dividing the IC_50_ of cholesterol ( = 100%) by the IC_50_ of each chemical.

### Ligand competitive binding assay of SlSCPx-2 mutants

The modeled 3-D structure of SlSCPx-2/AeSCPI-1 (Fig. 2B) and binding pocket analysis show that hydrophobic amino acid residues in the interior ligand-binding cavity are probably important for the protein-ligand interaction [19]. To confirm their roles in binding activity, several amino acid residues (F53, W66, F89, F110, I115, T128 and Q131) were mutated and their ligand-binding activity was tested (Table 5 and 6) with two representative ligands, cholesterol and palmitic acid, which had binding activity (IC_50_) values of 50.13 and 59.05 µM, respectively, for the wild type protein (Table 4). The results indicated that the mutants F53A, F53W, W66E, F89A, F110A, F110W and Q131A showed a decrease in binding activity to cholesterol compared to the wild type, while the mutants F53A, F53W, W66E, F89W, F110W, I115M and Q131A showed a decrease in binding activity to palmitic acid (Table 6). Replacing the Phe residue at position 53 with Trp (F53W) resulted in a dramatic loss of the cholesterol- and palmitic acid-binding activity. But F53A showed only a little bit decrease in palmitic acid-binding activity. Replacing the Trp residue at position 66 with Glu (W66E) remarkably lost its cholesterol-binding activity, probably due to the loss of the aromatic ring to form π-π stacking with cholesterol, but this mutant retained the palmitic acid-binding capacity. Replacing the Phe residue at position 89 with Ala (F89A) dramatically destroyed the cholesterol-binding activity. However, replacing the same residue with Trp (F89W) resulted in an increase in the cholesterol-binding activity. This may be because tryptophane can form more stable π-π stacking than phenylalanine, which facilitates the binding with sterols that have aromatic ring in its structure [2]. In contrast, the mutant F89A strongly increased the palmitic acid-binding activity. Replacing the Phe at position 110 with Ala (F110A) or Trp (F110W) decreased cholesterol-binding activity, especially for F110A, which lost cholesterol-binding activity completely. While F110A obviously increased palmitic acid-binding activity, F110W greatly decreased palmitic acid-binding activity. Replacing the Ile residue at position 115 with Met (I115M) lost its palmitic acid-binding activity completely, but increased its cholesterol-binding capacity. The T128 residue directly interacts with ligands (Fig. 2B), when this threonine was replaced with alanine, both the cholesterol and palmitic acid binding activities were increased, suggesting that it may control the entry of the ligands into the binding cavity. Q131 residue forms hydrogen bond with cholesterol. When the Q131 residue was changed into alanine, the ligand-binding ability was significantly decreased. All these results suggest that mutation of these individual amino acid residues could lead to changes in the ligand-binding activity and they are critical for the protein to bind with its ligands.

**Table 5:** The predicted amino acid residues in the secondary structure units that form the binding pocket of SlSCPx-2 protein.

Secondary structure unit	Amino acids in the binding pocket
α-Helix-1	Val25, Tyr28, Met29*
β-Sheet-I	Phe53*, Val55
β-Sheet-II	Trp66, Ile68
β-Sheet-IV	Phe89*, Val91, Ser92, Asp93
α-Helix-3	Val96, Val97, Leu99, Ile100
α-Helix-4	Pro106, Gln107*, Phe110
β-Sheet-V	Ile115, Ile117
α-Helix-5	Met121*, Ala124, Met125, Leu127, Thr128*, Leu130, Gln131*

These amino acid residues are predicted by using Pocket Finder as ligand binding sites, which directly or indirectly interact with the ligands.

**Table 6:** Changes in binding activity (IC_50_) of the SlSCPx-2 mutants to cholesterol and paltimic acid*.

Mutants	Cholesterol (µM)	RBA_chol_ (%)	Palmitic acid (µM)	RBA_pal_ (%)
Wild type	50.13±0.80	100	59.05±1.29	100
F53A	7300±280	0.7	71.72±0.84	82
F53W	4669±23.20	1	5777±28.02	1
W66E	974±10.72	5	67.33±3.43	88
F89A	1743±1.30	3	0.046±0.33	1.2×10^5^
F89W *	5.58±1.23	898	77.50±1.51	76
F110A	4.45×10^7^±7.6×10^5^	1.1×10^−4^	41.70±1.62	142
F110W	230.9±2.36	22	503.3±2.70	12
I115M *	34.30±1.53	146	6617±38.21	0.9
T128A *	20.82±1.32	241	32.82±1.52	180
Q131A	2694±34.20	1.9	354.8±2.55	17

IC_50_: concentration at 50% inhibition and the data are derived from assays described in Fig. 4B; RBA: relative binding affinity. RBA_chol_ and RBA_pal_: dividing the IC_50_ of wild type protein for cholesterol ( = 100%) or palmitic acid ( = 100%), respectively, by the IC_50_ of each chemicals.

### Virtual docking-based screening of ligands as inhibitors of SlSCPx-2

**Figure 5:**
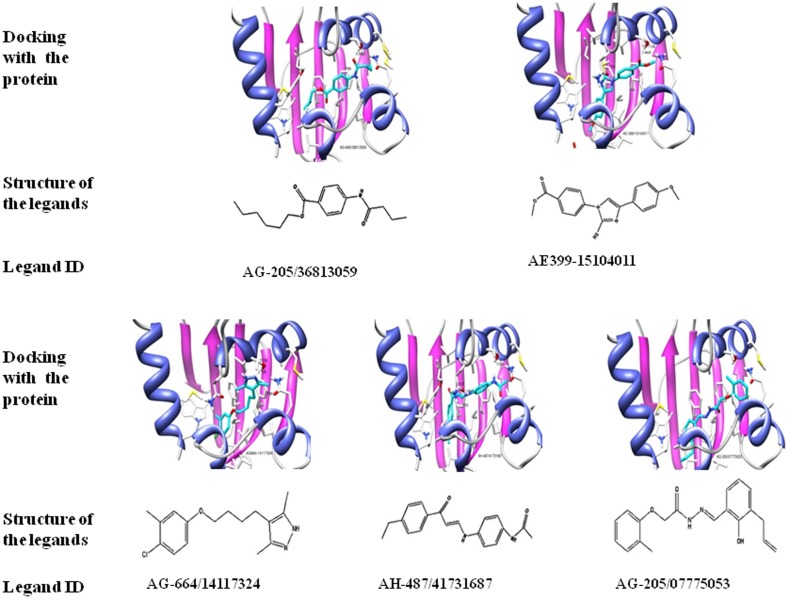
Fixness and structure of the 5 selected potential inhibitory compounds by virtual ligand screening based on the modeling structure of SlSCPx-2/AeSCPI-1 complex after MD optimization.

To obtain potential chemical inhibitors of SlSCPx-2, which can compete with the natural ligands, the compounds deposited in the SPECs Data Bank were screened. On the first step of 2-D ligand-based searching, the criteria in terms of the Lipinski rules were employed to preselect all the molecules in the SPECs database, generating 11,400 compounds for the subsequent docking-based screening. All preselected compounds were then transformed from 2-D to 3-D structure by using CONCORD module of SYBYL 7.3. A proper virtual screening cavity was generated based on the 3-D modeled structure of SlSCPx-2/AeSCPI-1 (Fig. 2B) and used for the docking-based screening. Two thousand sixty seven compounds were primitively selected after the Surflex-Dock processing. Subsequently, the top 100 compounds with the highest C-scores were selected to further analysis for potential specific inhibitors. The residues F53, T128 and Q131 of SlSCPx-2 were used as pharmacophore to screen these 100 compounds. The reason for using these three residues as key pharmacophore is that they are located in the binding pocket predicted by PoketFinder. The T128 and Q131 are at the center of a hydrogen bond network and can form hydrogen bonds with the AeSCPI-1 molecule (Fig. 2B). F53 is proximal to aromatic ring of SlSCPx-2 and may play a role in forming π-π stacking, which is important in ligand-binding [2]. By this screening strategy, 50 hit compounds with docking-scores higher than 5 were ultimately obtained (Table 7) [30]. Among these 50 compounds, 5 of them not only had high scores but also had suitable configurations for binding bonds between the protein and the ligands (Fig. 5). One (SPECS No. AH-487/41731687) of the compounds has a similar structure as AeSCPI-1 [20].

**Table 7:** Hit compounds selected from SPECS with high score values that potentially bind with SlSCPx-2 based on its modeled 3D-structure.

Specs ID	Total Score	Specs ID	Total score	Specs ID	Total score
AG-205/36868061	12.55	AG-690/40698645	10.95	AG-690/13416244	10.22
AG-205/33163045	12.43	AG-690/36813509	10.89	AF-399/13806043	10.21
AG-690/12890522	12.11	AG-690/40699342	10.88	AG-690/40750360	10.21
AH-487/42271849	11.63	AG-690/36933045	10.88	AG-690/12408430	10.19
AF-399/15105077	11.56	AG-690/11821369	10.77	AG-205/13381137	10.18
AF-339/42217087	11.48	AG-670/40949132	10.76	AF-399/4235635	10.17
AG-690/40696270	11.43	AG-690/15440485	10.72	AG-690/09289029	10.15
AH-487/41825275	11.41	AE-848/13008366	10.68	AG-690/40700936	10.13
AG-664/14117324*	11.37	AF-399/37418001	10.66	AG-205/07775053 *	10.10
AF-399/14183757	11.35	AF-399/12153029	10.54	AF-399/11380003	10.01
AG_205/40776077	11.34	AF-399/40862938	10.53	AH-487/40686394	9.92
AG-205/36813059*	11.27	AH-487/41731687*	10.52	AG-205/33005046	9.77
AF-399/41767570	11.22	AG-690/15442177	10.46	AE-399/15104011 *	9.65
AG-690/12885304	11.20	AG-690/15439252	10.43	AG-205/36953210	9.31
AH-487/40935616	11.16	AG-690/12885340	10.33	AG-641/12753010	9.05
AG-690/15435898	11.09	AG-205/04772018	10.31	AG-690/11571027	8.85
AG-690/40696907	11.06	AG-690/40696514	10.27		

These compounds were selected for the *in vitro* competitive binding assay and bioassay.

### Competitive binding assay and bioassays

**Figure 6:**
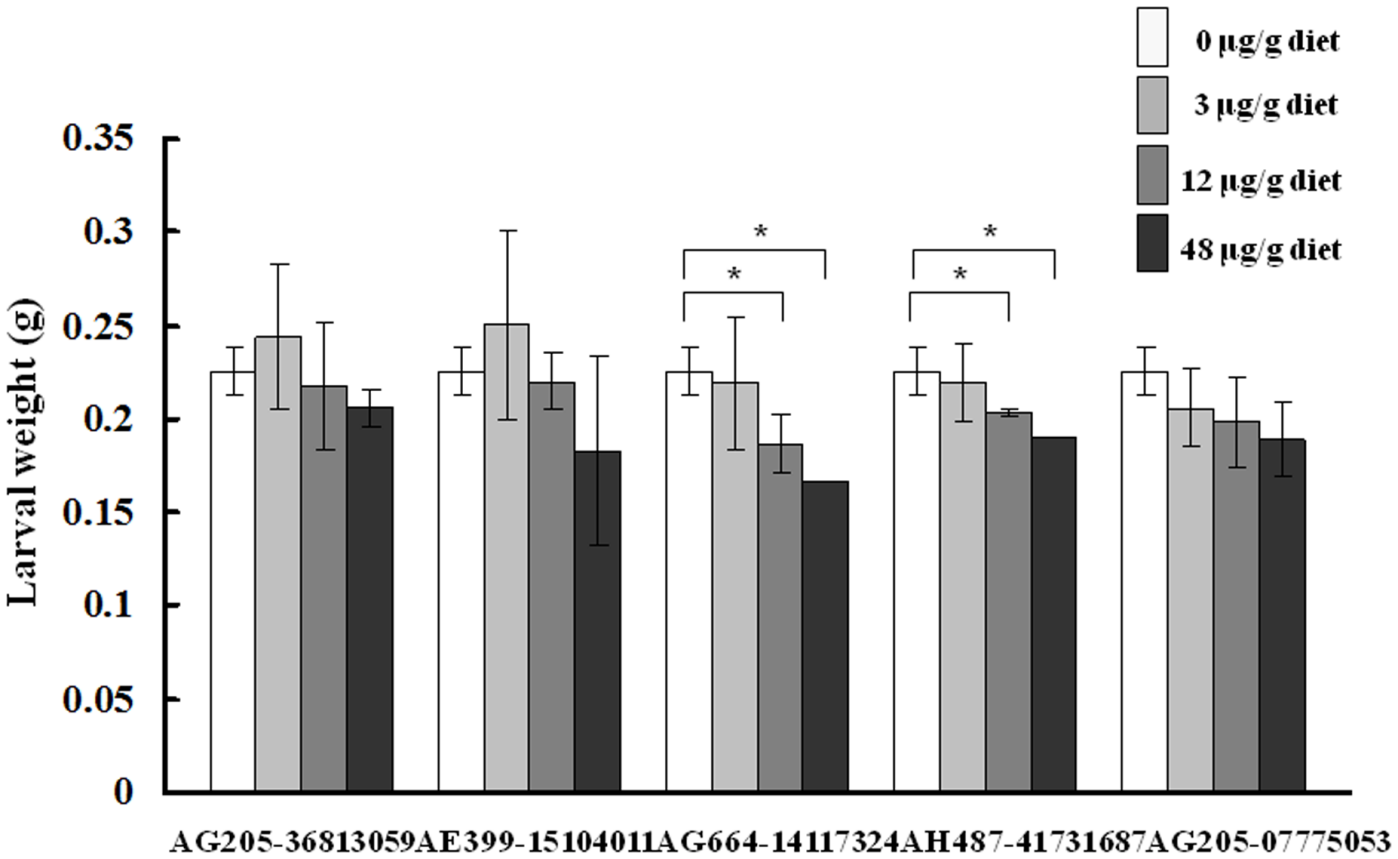
Bioassays of the selected compounds for inhibition of growth of *S. litura* larvae. Newly hatching larvae were treated with the individual compounds at different concentrations and the body weight of the larvae was recorded at the indicated time points. At 13 days post egg hatching, most of the larvae were at 5^th^ instar stage.

To assess the SlSCPx-2-binding and biological activity of potential chemicals screened by the Surflex-dock analysis, *in vitro* competitive binding assay and *in vivo* bioassays for five selected chemicals (AG-664/14117324, AH-487/41731687, AG-205/36813059, AG-205/07775053 and AE-399/15104011) were conducted. The results of the *in vitro* competitive binding assay showed that the compounds AG-664/14117324 and AH-487/41731687 had higher binding activity than cholesterol and AeSCPI-1, whereas the compounds AG-205/07775053 and AE-399/15104011 had lower affinities than cholesterol (Table 8). The results of the *in vivo* bioassays for five selected chemicals indicated that these compounds had different degrees of inhibitory activity to growth of the *S. litura* larvae (Fig. 6). With the increase in the treatment concentrations, the body weight at 13 days post egg hatching of the treated larvae decreased, significantly for AG-664/14117324 and AH-487/41731687, as compared to the control.

**Table 8:** Binding activity of selected compounds by the *in vitro* competitive binding assay.

Ligands	IC_50_ (µM)	RBA(%)
Cholesterol	50.13±0.80	100
AG-664/14117324	2.58±0.20	1943
AH-487/41731687	4.30±0.63	1166
AG-205/36813059	23.88±1.38	210
AeSCPI-1	160.9±5.03	31
AE-399/15104011	794.3±40	6
AG-205/07775053	8632±393	0.6

IC_50_: concentration at 50% inhibition. RBA: relative binding affinity, dividing the IC50 of cholesterol ( = 100%) by the IC_50_ of each chemicals.

## Discussion

In the previous study we found that over-expression of SlSCPx and SlSCPx-2 proteins in *S. litura* Spli-221 cells enhanced cholesterol uptake and knocking-down SlSCP*x* transcripts by dsRNA interference resulted in a decrease in cholesterol level in the hemolymph and delayed the larva-to-pupa transformation [25]. Because insects lack two key enzymes for *de novo* biosynthesis of cholesterol, they have to use exogenous cholesterol from their food for ecdysteroid synthesis and growth and development [3]–[5]. Targeting insect SCP-2 may be an effective approach for the pest control. Some reports have demonstrated that SCP-2 can bind different sterols and fatty acids [2], [10]. In this study, we modeled the 3-D structure of the SlSCPx-2 protein and examined its binding affinity and specificity against different sterols and fatty acids by using a combining strategy of structural analysis and compound-binding assay.

The results indicated that SlSCPx-2 protein could bind to both sterols and fatty acids (Table 2–4, Fig. 4), many of which are natural compounds in the host plants that *S. litura* feed on, such as stigmasterol, cholesterol and β-sitosterol. It has been found that AeSCP-2 in *A. aegypti* can not only bind cholesterol but also fatty acids [2]–[3]. The protein has higher binding affinity to cholesterol than to fatty acids [2], [33]. Other two SCP-2 like proteins in *A. aegypti*, AeSCP-2L1 and AeSCP-2L2, also showed different binding preference to sterols and fatty acids. AeSCP-2L1 has higher affinities to fatty acids than sterols, whereas AeSCP-2L2 has higher affinities to sterols than fatty acids [11]. Vertebrate SCP-2 proteins also can bind sterols and fatty acids with the following order of binding affinity: cholesterol>straight chain fatty acids>kinked chain fatty acids [10]. That a single protein can bind to different ligands with various structures would allow the protein to efficiently transport different substrates to meet the sterol and fatty acid requirement for growth and development of insects. It may be particularly important for polyphagous insects such as *S. litura* that has only a single gene of SCPx and needs to uptake and transport multiple sterols and fatty acids from its host plants.

To investigate the ligand-binding activity and mechanism of SlSCPx-2, the direct and best approach is to obtain its crystal structure and then study the relationship between structure and function. To date, crystal structures of several SCP-2 family members have been reported, including *T. thermophilus* TtSCP-2 (2CX7) [14], *P. cryptogea* PcSCP-2 (1LRI) [15], *A. aegypti* AeSCP-2 (1PZ4), AeSCP-2L2 (2QZT) and AeSCP-2L3 (3BKR) [6], [9], [16], *H. sapiens* HsSCP-2 (2COL) and HsMFE-2 (1IKT) [17]–[18], and *O. cuniculus* OcSCP-2 (1C44) [19]. However, no crystallographic structure of lepidopteran SCPx-2 has been reported. Sequence alignment suggests that *S. litura* SCPx-2 was structurally more similar to HsMFE-2 (Fig. 1), which was bound with non-natural detergent TritonX-100 within its hydrophobic cavity [18], than to the dipteran AeSCP-2, which bound with palmitic acid [9]. Therefore, in this study, HsMFE-2 with a TritonX-100 molecule in its binding pocket was first used as a template for the modeling of the SlSCPx-2 3-D structure. The results indicate that the resultant basic 3-D structure of SlSCPx-2 is effective and suitable for the most of the compounds tested in this study. This is also experimentally confirmed by fluorescence replacement assays with different fatty acids and sterols.

In AeSCP-2, the head of palmitic acid interacts with a loop that connects the first helix and the first β-strand, whereas in HsMFE-2, as well as OcSCP-2, this loop is replaced by a short α-helix, which makes the mammalian proteins to coordinate the carboxyl moiety of fatty acids in a different manner from the insect proteins [16]. In addition, the palmitic acid in AeSCP-2 lies in the vertical direction with several arginines to stabilize the ligand-binding activity, while TritonX-100 in HsMFE-2 lies in the horizontal position with the polyoxyethylene tail extending in the opposite direction away from the carboxyl group of the fatty acid. The mosquito AeSCP-2L2 possesses the ability to bind natural ligands in both the vertical and horizontal orientations because it can form a dimmer to accommodate three natural palmitic acids [16]. The horizontal direction of ligands in SlSCPx-2 is similar to that in the HsMFE-2, other than to that in insect proteins, in which ligands lie in vertical direction.

Virtual ligand screening results show that sterols have relatively lower binding scores, as compared to fatty acids. This may be due to that sterols can form only hydrophobic interactions with SlSCPx-2. However, for the lipids, especially for those 14–16C and 20–22C fatty acids, they can form stable hydrogen bonds with SlSCPx-2, and therefore have higher binding scores than the sterols. The configuration of the lipids and sterols that have high docking scores can provide some clues for the computational screening of the potential inhibitors. In this study, 5 compounds were finally selected by this strategy (Fig. 5) and their inhibitory effects on growth of larvae were confirmed.

AeSCP-2 mutation assays showed that the mutations of F9W, W44F, F105A, F105W, F32A, F32W, W44E and M90L decrease the binding activity of the protein with NBD-cholesterol, as compared to the wild type [24], suggesting that these amino acids play an important role in the interaction between the protein and ligands. Therefore, in the present study, the amino acid residues F53, W66, F89, F110, I115, T128 and Q131 in SlSCPx-2, which may contribute to the ligand binding pocket, were mutated and tested for their roles in ligand binding activity (Table 6). The F89W mutant strongly increased the ability of binding with cholesterol, but decreased the ability of binding with palmitic acid. This may because that tryptophane can form a more stable π-π stacking than phenylalanine to stabilize the binding ligand cavity [2], which favors for cholesterol binding. W66 and F110 can be used not only to stabilize the binding cavity so that it could accommodate different sizes of ligands but also to form π-π stacking bonds with the ligands. When Trp at the position of 66 was changed into Glu or Phe at the position of 110 was changed into Ala/Trp, their cholesterol-binding activity was greatly decreased. Based the modeled structure, T128A increased both cholesterol and palmitic acid binding activity. T128 may directly interact with the bound ligand by forming hydrogen bonds at the entrance site of the binding pocket. Mutation of this residue would lead to lose of the hydrogen bond, allowing the ligand more easily to enter the pocket, thus increasing the binding activity with cholesterol and palmitic acid. F53 and F89 can form the π-π stacking bonds with the aromatic ring of AeSCPI-1 [2]. When F53 was changed to A or W, they lost the cholesterol binding ability and reduced palmitic acid binding activity. However, when F89 was replaced by Ala, its binding affinity to cholesterol was greatly reduced, but its binding affinity to palmitic acid was dramatically increased. When this residue was changed to Trp, its affinity to cholesterol was increased greatly. I115M lost the palmitic acid binding activity, while the mutant increased cholesterol binding capacity. This result is similar to the finding in *E. lagascae* SCP-2, where a single Leu/Met exchange alters lipid/sterol binding activity [23]. When it is a Leu, it favors to bind with fatty acids, while Leu is changed into Met, it favors to bind with cholesterol. T128, which lies at the entrance site of the binding cavity, could directly interact with AeSCPI-1. Mutation of this residue would lead to lose of the hydrogen bond, allowing the ligands more easily to enter the cavity. Q131 can form a hydrogen bond with the ligands and the loss of the bond would obviously decrease both the paltimic acid- and cholesterol-binding activity. These results suggest that F53, W66, F89, F110, I115, T128 and Q131 are critical for the ligand binding affinity and specificity and proper residues in the binding cavity are required for stable binding with the ligand subtracts.

Five chemicals were chosen from those screened by SPECS (Table 7) to check their competitive binding activity against AeSCPI-1 and biological activity of inhibition of growth of *S. litura* larvae. Three of these chemicals (AG-664/14117324, AH-487/41731687 and AG-205/36813059) had higher affinities to SlSCPx-2 than AeSCPI-1. AG-664/14117324 and AH-487/41731687 had higher affinities than cholesterol (Table 8). This result is consistent with biological activity (Fig. 6). These two compounds have a carbon chain connecting two aromatic ring. AG-205/36813059 has only one aromatic ring in its configuration (Fig. 5) and also showed a certain degree of biological activity (Fig. 6). AG-205/07775053 also had a high inhibitory effect on the larval growth, but its affinity was lower than AeSCPI-1. AF-399/15104011 has three aromatic rings and has lower binding affinity to the protein and biological activity. These results indicated that the activity of inhibiting the growth of larvae may be partially related to the numbers and configuration of the aromatic rings in the compounds.

## Conclusions

Virtual screening and ANS fluorescence displacement assays showed that SlSCPx-2 can bind with sterols and fatty acids. The protein has higher affinities to fatty acids than to sterols. Site-directed mutation of SlSCPx-2 suggests that F53, W66, F89,IF110, 115, T128 and Q131 are the critical residues for the interaction between the protein and ligands. SPECS screening suggested several hit lead compounds may have potential for being used as SlSCPx-2 inhibitors. Bioassay indicated that AG-664/14117324, AH-487/41731687, AG-205/36813059 and AG-205/07775053 had inhibitory effect on the growth of *S. litura* larvae.
